# Elucidation of the Initial Growth Process and the Infection Mechanism of *Penicillium digitatum* on Postharvest Citrus (*Citrus reticulata* Blanco)

**DOI:** 10.3390/microorganisms7110485

**Published:** 2019-10-24

**Authors:** Xin Qian, Qiya Yang, Qidi Zhang, Mandour H. Abdelhai, Solairaj Dhanasekaran, Boateng Nana Adwoa Serwah, Ning Gu, Hongyin Zhang

**Affiliations:** 1 School of Food and Biological Engineering, Jiangsu University, 301 Xuefu Road, Zhenjiang 212013, China; 2 Nanjing Dongshan Foreign Language School, 99 Shanggao Road, Jiangning District, Nanjing 211103, China

**Keywords:** *Penicillium digitatum*, citrus, initial process, cell wall degrading enzyme, RNA-seq

## Abstract

Green mold disease, a common citrus post-harvest disease caused by *Penicillium digitatum*, has an unresolved initial infection mechanism. Understanding the infection mechanism leads to the development of potential controls and preventive measures against the disease. The present study aimed to delineate the infection mechanism by investigating spore germination, changes of organic molecules and enzyme activity, and differential expression of genes in the *P. digitatum* infection. *P. digitatum* spore germination was observed by a pathology section scanner and it was found that in vivo germination was 3 h behind the in vitro germination. In addition, cell wall degrading enzymes and soluble sugar and titratable acid content during the infection process measured dynamically. The level of pectinase reached its maximum of 6067 U/g before 48 hpi, while cellulase increased rapidly after 48 hpi. The soluble sugar and organic acid content increased considerably with the progression of the infection. The transcriptomic profile of *P. digitatum* before and after infection was analyzed by RNA-seq. The genes related to cell wall degrading enzymes were significantly up-regulated and annotated to participate in two major carbon source synthesis pathways. The study delineated the initial infection mechanism of *P. digitatum* which eventually opened the gate way for the development of new control strategies in the future.

## 1. Introduction

Citrus fruits grow on flowering trees and shrubs, and are known as one of the four big fruits of the world. Citrus is delicious in taste and is a rich source of ascorbic acid and dietary fiber [1]. However, it undergoes significant economic losses due to rot rate of as high as 50% during post-harvest transportation [2]. Post-harvest green mold disease caused by *P. digitatum*, is the most serious infection of citrus [3]. Currently, the use of chemical fungicides is the common method of preventing and controlling post-harvest green mold infections, though its safety has been criticized [4]. Some molds like *P. digitatum* were reported to have the ability to become resistant against common fungicides, so there is a growing demand for safe and effective alternative control methods [5].

Pathogen invasion into plant tissue or cells is a complex process in which the pathogens need to break several barriers like plant epidermal tissue, cell wall and the immune system of plant cells [6]. Green mold caused by *P. digitatum* was reported in high diversified fruits, but their parasitic ability is weak. In most cases the pathogen infects the fruit and lurk on the surface, but does not affect the fruit quality. *P. digitatum* invade into the tissue only when the tissues of the fruit are bruised or wounded [7]. After successful invasion into the host tissues, the pathogen requires optimum conditions such as temperature and humidity to grow and reproduce. The optimum temperatures of most *Penicillium* strains are between 23 °C and 25 °C. The most notable *Penicillium* spps. like *P. italicum* grow optimally at 23 °C, and the optimum growth temperature of *P. digitatum* is 25 °C which is sparingly greater [8].

Though the pathogens enter into the host and have suitable environment for their growth and proliferation, the cell wall of the host acts as a primary defense barrier to reduce pathogen attacks [9]. The basic components in the cell wall of plant systems includes cellulose, hemicellulose, pectin and structural protein [10]. According to Kubicek et al. [11], most pathogens invade host tissues by secreting cell wall degrading enzymes (CWDEs). The CWDEs are classified as pectinase, cellulase and hemicellulase according to their various degradation substrates. Pectinase is a group of inducible complex enzymes which have the ability to degrade pectin or pectic acid to galacturonic acid [12]. Pectin hydrolases can cause fruit softening and decay by degrading the pectin in the intercellular layer [13]. Cellulases and hemicellulases released after the plant tissue structure become relaxed and expand the area of infection further. Cellulases can break down cellulose molecules to glucose by hydrolyzing the beta-1,4-glucosidic bonds [14]. Cellulase is the group of enzymes including exo-1,4-beta-glucanase (Cx), endo-1,4-beta-D-glucanase (C_1_) and beta-glucosidase (β-G) [15]. Similarly, the citrus peel is a rich source of pectin and cellulose. However, to the best of our knowledge, there is little information available on whether and how CWDEs play a key role in the postharvest infection of *P. digitatum* of citrus.

The exploration of the pathogen infection process into plant tissue through microscopic observations can give a deeper understanding of pathogen infection mechanisms. A few examples of such observations were reported by Zhao et al. [16] where they observed the early infection process of *Colletotrichum fragariae* in strawberry leaves with fluorescent markers. In addition, Yuan et al. [17] studied *Fusarium oxysporum* infected strawberries by scanning electron microscopy, and further explored the interaction mechanism between pathogen and host. However, no article has been found on the microscopic observation of the postharvest infection of *P. digitatum* on citrus. Microscopic observation can bring an intuitive understanding of the infection process of pathogens, and correlated with findings from some other infection mechanisms of the pathogen, also identify key time points. However, the infection mechanism of *P. digitatum* in citrus fruits is not fully understood, therefore, it was of great significance that we studied the infection process of *P. digitatum* and the mechanisms involved at the physiological and molecular levels. 

In the present study: (1) The early infection process of *P. digitatum* in citrus wound tissues was investigated by sectioning the tissues pathology section scanner. (2) The activity of the main cell wall degrading enzymes, soluble sugar and titratable acid contents of citrus fruits during the process of infection was measured dynamically. (3) The key time of early infection process of *P. digitatum* was obtained based on the results of above-mentioned experiments. The transcriptomic profile of *P. digitatum* before and after the infection was analyzed by RNA-seq. Subsequently, we focused on the expression of genes related to cell wall degrading enzymes and their metabolic pathways. This study provides a good knowledge of elucidation about infection mechanism of *P. digitatum* on postharvest citrus during initial infection process.

## 2. Materials and Methods

### 2.1. Pathogen and Fruit

The pathogen was isolated from natural rotten citrus and identified as *P. digitatum* by sequencing the 5.8S internal transcribed spacer (ITS), beta-tublin (BenA) and Calmodulin (CaM) DNA regions and sequence similarity search in NCBI GenBank [18]. Spore suspensions were prepared from the 7-day-old *P. digitatum* culture in sterile saline and were adjusted to an appropriate concentration (1 × 10^6^ spores/mL) using a hemocytometer [19].

Citrus fruits (*Citrus reticulata* Blanco) of similar color and maturity stage that were free of any visible mechanical damage were selected from an orchard, and further used for experiments [20].

### 2.2. Infection of Citrus by P. digitatum

Three identical wounds (3 mm width × 5 mm deep) were created on the equator of the citrus fruits after washing and sterilizing in 1% sodium hypochlorite. 30 µL of the *P. digitatum* suspension was inoculated into each wound. Samples were stored at optimal conditions (25 °C, 95% relative humidity). The decay incidence and lesion diameter of the treated citrus fruits were recorded every 12 h [21]. Three replicates per treatment were performed with 36 citrus fruits for each treatment (thus 12 citrus fruits per replicate). This experiment was performed three times.

### 2.3. The Growth Process of P. digitatum in Vitro

An amount of 2.5 µL of the spore suspensions of *P. digitatum* was spread on the surface of the PDA plates and cultured at 25 °C, and 95% relative humidity. Tissues of samples (treated portions of the citrus fruits) were excised with a scalpel and stained with carbonic acid lactate cotton blue stain for 5 min. Neutral glue was used to fix it to a slide. After air-drying, the excess neutral glue on the edge of the cover slide was cleaned with xylene and the infection process was observed at different time intervals (0 hpi, 3hpi, 6 hpi, 9 hpi, 12 hpi, 20 hpi, 28 hpi, 36 hpi and 44 hpi) with a panoramic MIDI slide scanner. There were three replicates per treatment, and the entire experiment was performed three times.

### 2.4. The Process of P. digitatum Infection of Citrus Fruits

Preparation and observation of citrus wounds were done as described above in Section 2.2 and Section 2.3, respectively. Three replicates per treatment were performed and this experiment was carried out three times.

### 2.5. Enzyme Activity Assay

The method used in conducting this experiment was a slightly modified version of that described by Mendes [22].

To measure the activity of enzymes secreted by *P. digitatum*, a 5 g of the peel was taken from the junction of healthy and diseased on 5 citrus wounds with a sterilized scalpel. An amount of 5 mL of the extraction buffer (1 mol/L NaCl, 20 mmol/L Tris-HCl, pH 7.4) was added, and ground in a cold mortar to form a homogenate. More of the extraction buffer was added to make up a 20 mL solution. After shaking thoroughly, it was centrifuged at 18353 × *g* at 4 °C for 20 min and the supernatant used as extract for enzyme assay.

The reaction systems of the enzyme β-glucosidase (β-G) was created as follows: 0.5 mL crude enzyme extract was added to 1 mL 50 mmol/L citric acid-sodium citrate buffer (pH 5.0), 1.5 mL 1% cellobiose solution (prepared with citrate buffer, pH 4.5) and incubated at 50 °C for 60 min in water bath. After 60 min, 3 mL of 3,5-dinitrosalicylic acid reagent (DNS) was added to stop the reaction. Then, the mixture was boiled for 5 min and immediately cooled under running water. To measure the activity of the enzyme, distilled water was added until 15 mL and then the absorbance was measured at 540 nm. The enzyme activity unit (U/g) was defined as the amount of enzyme required to produce 1 µg of glucose per hour at 50 °C. Cellobiose solution was added to the control group after the addition of the DNS reagent. 

The assay preparation procedure for the activity of Exo-1,4-β-glucanase (Cx) was the same as β-G except that the substrate was 1% sodium carboxymethyl cellulose (CMC).

The activity of polygalacturonase (PG) was determined as follows: To 0.5 mL of crude enzyme extract, 1 mL of 50 mmol/L sodium citrate buffer (pH 5.0), and 1.5 mL of 1% polygalacturonic acid solution (formulated with citric acid buffer, pH 4.5) was added. After incubating at 50 °C for 60 min in water bath, 3 mL of DNS reagent was added to stop the reaction. Then, the mixture was boiled for 5 min and cooled under running water. To measure the activity of the enzyme, distilled water was added until 20 mL and then the absorbance was measured at 540 nm. The enzyme activity unit (U/g) was defined as the amount of enzyme required to produce 1 µg of glucose per hour at 50 °C. Polygalacturonic acid solution was added to the control group after the addition of the DNS reagent.

The preparation of the reaction systems for the determination of pectin methylgalacturonase (PMG) activity was similar to that of PG except that the substrate was 1% pectin. 

Each treatment was done in three replicates and the entire experiment was repeated three times.

### 2.6. Effects of P. digitatum on Biochemical Changes of Citrus Wound Tissues

The experiment was done by slightly modified version of the method described by Zeng et al. [23].

A total of 5 g of tissue with peel was taken from five citrus wounds and ground with 10 mL distilled water. The homogenate was transferred into a centrifuge tube and centrifuged at 4000× *g* for 20 min at 4 °C after 1 h at room temperature. The supernatant was taken at a constant volume of 100 mL. After 30 min of boiling in water bath, it was cooled and filtered.

To determine the titratable acid contents of the treated samples, 1% phenolphthalein indicator was added to 10 mL of the above extract and titrated against a calibrated sodium hydroxide solution. Distilled water was used instead of the filtrate/extract for titration in the control group.

To quantify the soluble sugar contents of the sample, 10 mL of the above extract was taken and diluted to 50 mL. 0.5 mL of anthrone reagent and 5 mL of concentrated sulfuric acid was added into 2 mL of the diluted extract in a test tube. Then the absorbance was measured at 630 nm. Distilled water was used instead of the filtrate/diluted extract for the soluble sugar measurement for the control group.

### 2.7. Transcriptome Assay

#### 2.7.1. Sample Preparation

Preparation of fruit and *P. digitatum* inoculation was done as described above (Section 2.3). After 44 hpi, the mycelia collected from the infected fruit was used as treated group sample, and *P. digitatum* grown in suspension culture in PDB medium was used as the control. There were two replicates per sample.

#### 2.7.2. Total RNA Extraction

To prepare transcriptome, total RNA was extracted using the fungal RNA extraction kit according to the operation instructions of Sangon. Co., (Shanghai, China). RNA purity, concentration and integrity were determined with reference to Hsu et al. [24].

#### 2.7.3. RNA-Seq Library Construction and Sequencing

Prior to using mRNA as a template, the eukaryotic mRNA was enriched with magnetic beads with Oligo (dT), the fragmentation buffer was used to randomly break the mRNA. Secondly, the first cDNA strand was synthesized with random hexamers, then a second cDNA strand was synthesized by adding buffer, dNTPs, RNase H and DNA polymerase I. The purified double-stranded cDNA was repaired with A-tailed and ligated to the sequencing linker, and fragment size selection was performed using AMPure XP beads. Finally, a cDNA library was obtained by PCR enrichment. After the library was constructed, preliminary quantification was performed using Qubit 2.0. The library was diluted, and then Agilent 2100 was used to detect the insert size of the library. After the insert was found to be in accordance with expectations, the effective concentration of the library was accurately quantified using RT-qPCR. After the library was tested, different libraries were pooled to the flow cell according to the effective concentration and the target data volume. The cBOT was then clustered and sequenced using the Illumina high-throughput sequencing platform (HiSeq/MiSeq) [25]. The transcriptome data was submitted to the Gene Expression Omnibus (GEO) database, and numbered as GSE128979.

#### 2.7.4. De Novo Assembly and Bioinformatics Analysis of RNA-seq Data

Raw data was filtered to obtain high quality and clean data. The clean data was sequence aligned with the specified reference genome (GCF_000315645.1_PdigPd1) to obtain a mapped data. DESeq2 was used to analyze the differential expression level of each gene in two samples (|log2(Fold Change) | ≥ 1 and false discovery rate (FDR) < 0.05 were used as screening criteria). The significance of the p-value of the original hypothesis test was corrected using the accepted Benjamini–Hochberg correction method. 

### 2.8. Statistical Analysis

The data were analyzed for significant differences by analysis of variance (ANOVA) with the Minitab statistical package (version 17), using the Tukey test at *p* < 0.05. Results were presented in tables and figures.

## 3. Results

### 3.1. The Infection of Citrus by P. digitatum

**Figure 1:**
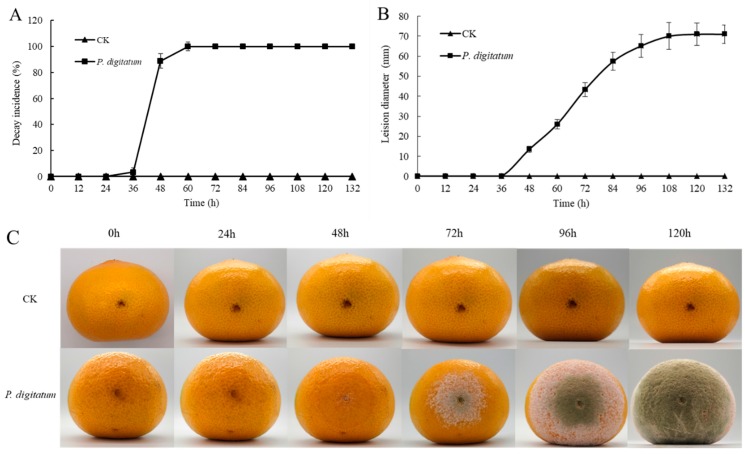
Infection of citrus by *P. digitatum* at 25 °C. (**A**): Decay incidence of citrus inoculated with *P. digitatum*; (**B**): lesion diameter of citrus inoculated with *P. digitatum*; (**C**) development of watery soft rot and green mold symptoms on citrus inoculated with *P. digitatum* in the same citrus. CK: Citrus inoculated with water. Each value is the mean of three experiments. Bars represent the standard deviation of the mean.

As shown in Figure 1A the citrus fruits inoculated with *P. digitatum* at 25 °C recorded a significant increase in rot at 48 h post inoculation (hpi) compared to the control sample, and the decay incidence was observed increasing rapidly and reached 100% at 60 hpi. At this time, the diameter of the decay was about 26.02 mm. An extension in culture time, revealed a rapid expansion of the diameter of the decay. The whole fruit was decayed (69.78 mm) at 108 hpi, unlike the control group which was not showed any decay (Figure 1B). Moreover, in the observation of the surface morphology of citrus fruits, inoculation of *P. digitatum* into wounds resulted in tissue maceration after 48 hpi and the tissue maceration nearly spread to the whole fruit at 96 hpi. A small number of white mycelia green colonies was found on the wound. At 132 hpi, the surface of citrus fruits was completely decayed and the wounds formed flaky colonies, with green mature fungi at the center and white mycelium at the outer edge (Figure 1C).

### 3.2. The Growth Process of P. digitatum on PDA

**Figure 2:**
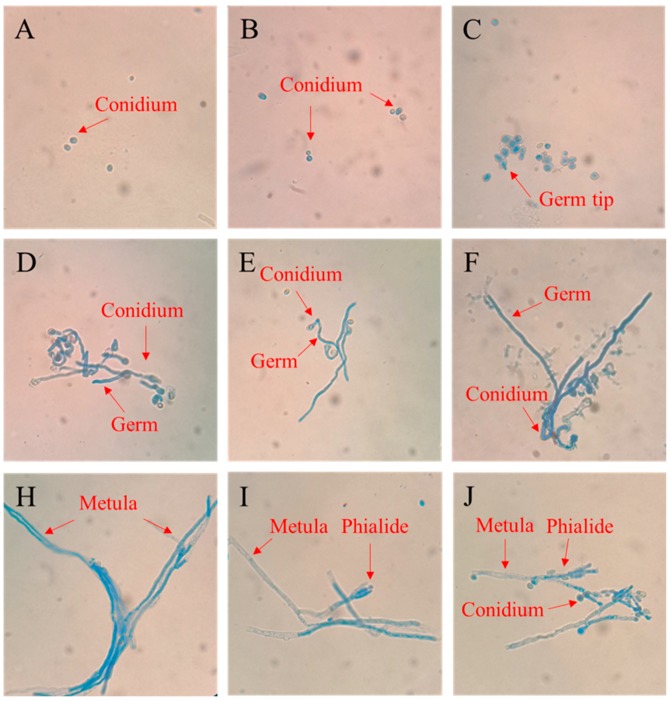
Observation of the growth process of *P. digitatum* on PDA at 25 °C. (**A**–**C**) Germination of *P. digitatum* spores at 0 h, 3 h, 6 h; (**D**–**F**) growth of *P. digitatum* in PDA at 9 h, 12 h, 20 h; (**H**–**J**) growth of *P. digitatum* in PDA at 28 h, 36 h, 44 h. Scale bar represents 20 µm, and is applicable to (**A**–**J**).

The results indicated that the spores of *P. digitatum* grew rapidly after it was inoculated onto the PDA medium, there was no apparent change in spores from 0 to 3 hpi, but it swelled obviously at 6 h, and a few spores began to show bud tips (Figure 2A–C). Nearly all spores germinated at 9 hpi, and the germ tubes grew gradually between 9 and 20 hpi (Figure 2D–F). In the field of spore growth for 28 hpi, the germ tube developed into a thick mycelium (Figure 2H), and phialide grew at the end of the mycelium at 36 hpi. With the extension of culture time to about 44 hpi, free conidia were found around the mycelium.

### 3.3. The Growth Process of P. digitatum in Citrus Wounds

**Figure 3:**
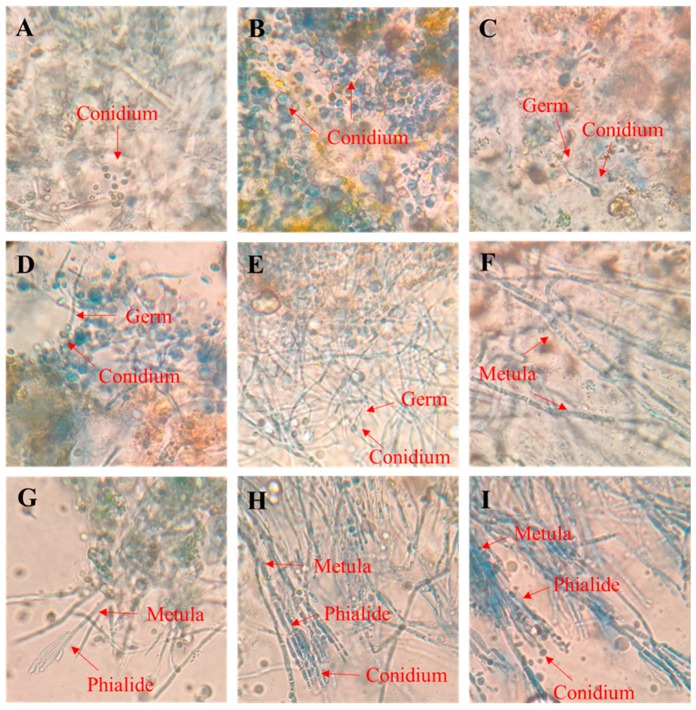
Observation of *P. digitatum* penetration on wounded citrus tissue at 25 °C. (**A**–**C**) Citrus tissue inoculated with *P. digitatum* spores for 0 h, 6 h, 9 h; (**D**–**F**) Growth of *P. digitatum* spores in citrus wounds after 12 h, 20 h, 28 h; (**G**–I) growth of *P. digitatum* spores in citrus wounds after 36 h, 44 h, 52 h. Scale bar represents 20 µm, and is applicable to (**A**–**I**).

*P. digitatum* spores swelled significantly after 6 hpi in the wounds of citrus fruits and few spores even begun to germinate in the field of vision after 9 hpi (Figure 3A–C). Most spores had germinated tubes at 12 hpi and all spores had developed into long germinating tubes after 20 hpi. At 28 hpi, thick mycelia were observed in the wound (Figure 3D–F). After prolonging the infection time to 36 hpi, the results showed a small number of prototypes of the phialide at the edge of the field (Figure 3G). At 44 h, a mature Phialide was observed at the end of the Metula, and conidia was observed on Phialide (Figure 3H). In the field of spore growth, the conidia on phialide were observed to have fallen off and laid freely near the mycelium 52 hpi (Figure 3I).

### 3.4. Effects of P. digitatum on Cell Wall Degrading Enzyme Activity of Citrus

**Figure 4:**
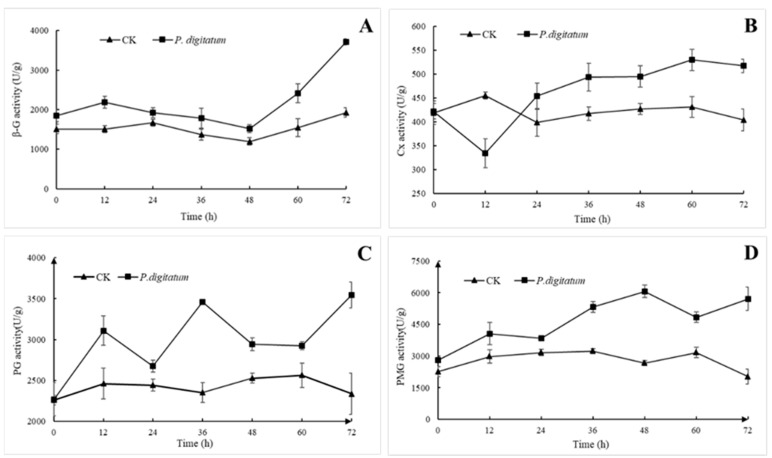
Effects of *P. digitatum* on cell wall degrading enzyme activity of citrus wound tissues at 25 °C. (**A**): Beta-glucosidase (β-G) activity; (**B**): exo-1,4-beta-glucanase (Cx) activity; (**C**): polygalacturonase (PG) activity; (**D**): pectin methylgalacturonase (PMG) activity. CK: Citrus inoculated with water. Each value is the mean of three experiments. Bars represent the standard deviation of the mean.

After *P. digitatum* was inoculated into citrus fruit wounds, the activities of PG and PMG were significantly higher than those of the control group, and the overall trend comparatively increased with progression in time. The activity of Cx was slightly inhibited at the initial stage and increased steadily after 24 hpi (Figure 4). Comparatively, the activities of PMG and PG belonging to the pectinase were higher, than that of the cellulose group (β-G and Cx) though the overall activity of Cx was much lower than those of the other three enzymes irrespective of group category.

As illustrated in (Figure 4A), the activity of β-G in the wounds of citrus fruits inoculated with *P. digitatum* was slightly higher than that of the control group between 0 to 48 h, but there were no significant differences in them. The activity of β-G in the treated group then increased rapidly to 2416 U/g and 3709 U/g at 60 h and 72 h, respectively, which is 1.65 and 1.94 times that of the control group.

In Figure 4B, the results indicated that the dynamic changes of Cx activity in the wound of citrus fruits decreased significantly (21%) and reached the minimum point after 12 h of inoculation with *P. digitatum*, and then increased slowly till it reached the maximum peak of 530 U/g at 60 h. The Cx activity of the control group remained between 400–450 U/g, which was a little close to that of the experimental group.

The PG activity of citrus fruits treated with *P. digitatum* reached peaks at 12 hpi (3110 U/g), 36 hpi (3463 U/g) and 72 hpi (3546 U/g), which were 1.36 times, 1.57 times and 1.61 times that of the control group, respectively. There were no significant changes in activity of samples in the control group (Figure 4C). Nevertheless, at 72 hpi dispersion of values was higher, as shows by standard deviation.

PMG activity in citrus fruit wounds inoculated with *P. digitatum* increased steadily, and reached a peak of 6067 U/g at 48 hpi after which it declined slightly and further picked up the rise at 72 h. In comparison to activity values of samples in the control group it was 2.37 and 2.92 times higher, respectively. The control group maintained a stable and low activity state (Figure 4D).

### 3.5. Effects of P. digitatum on Biochemical Changes of Citrus Wound Tissues

**Figure 5:**
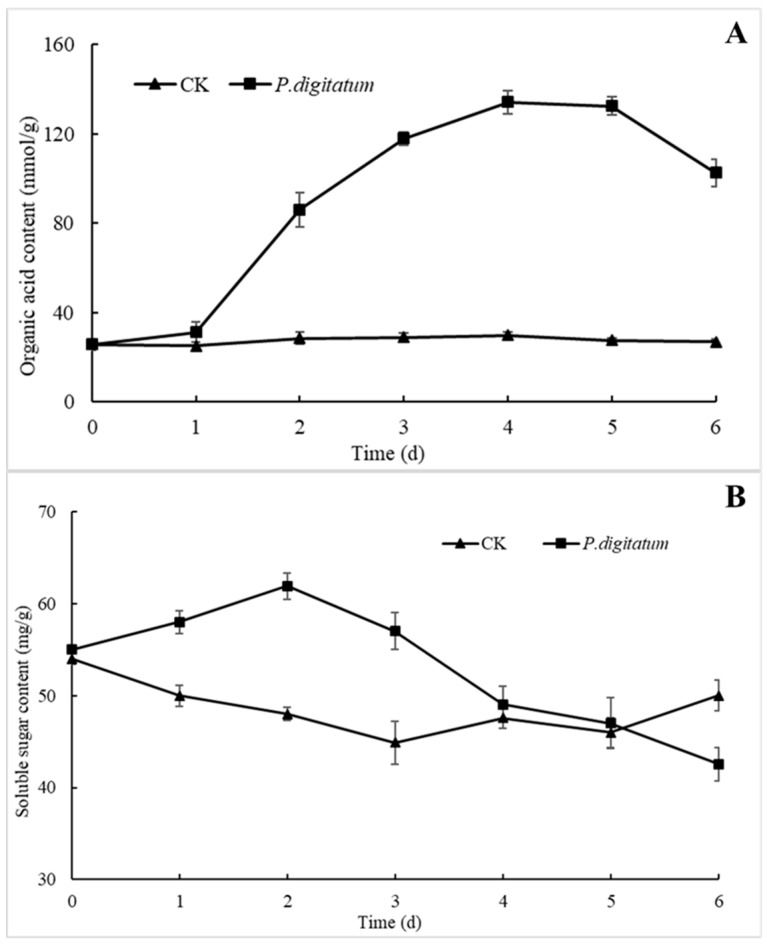
Effects of *P. digitatum* on biochemical changes of citrus wound tissues at 25 °C. (**A**): Organic acid; (**B**): soluble sugar. CK: Citrus inoculated with water. Bars represent the standard deviation of the mean.

The organic acid content in citrus fruit wounds infected by *P. digitatum* increased slowly on the first day, but increased rapidly in the next three days and reached the highest value (134.25 mmol/g) on day four, after which it declined slowly (Figure 5A). Meanwhile, the content of organic acid in the control group was almost unchanged.

The soluble sugar content in the treated fruits increased rapidly in the first two days, reached a maximum of 62 mg/g on day two, and then decreased continuously, leaving only 43 mg/g on day six, though the contents of soluble sugar in the control group was consistent at about 48 mg/g, and did not change much (Figure 5B).

### 3.6. Transcriptomic Analysis of P.digitatum Before and After Infection of Citrus

A total of 44.48 Gb clean data were obtained after transcriptome sequencing. The Clean Reads of *P. digitatum* (0 h) before infection were 24285615 and 398363318, and the GC Content were 54.46% and 54.51%, respectively. However, clean reads of *P. digitatum* (44 h) after inoculation into the wounds of citrus fruits were 32,810,982 and 51,322,479, and the GC Contents were 54.39% and 54.47%, respectively. In terms of statistical evaluation of base mass values, the Q30 base percentage of the four groups of samples reached above 94.39%.

**Figure 6:**
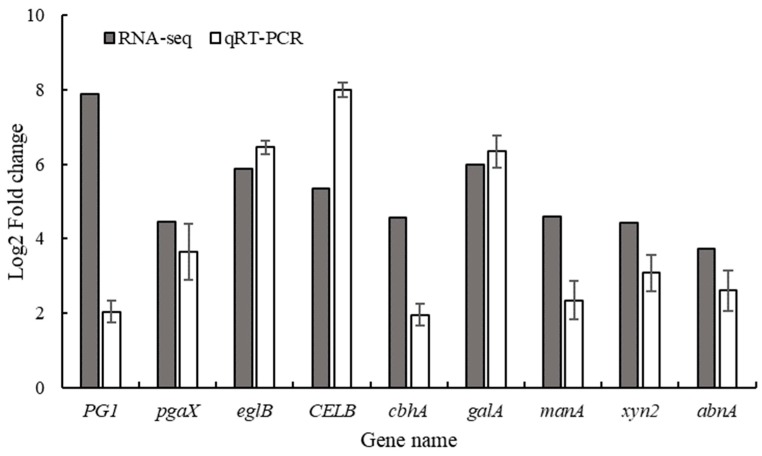
qRT-PCR validation for differentially expressed genes related to cell wall degrading enzymes.

In addition, the differentially expressed genes (DEGs) were functionally annotated with databases such as gene ontology (GO), SwissProt and Kyoto Encyclopedia of Genes and Genomes (KEGG). From the results we have found that some genes were related to CWDEs. Out of the lot (≥70 genes), that were annotated to participate in the synthesis of CWDEs in the SwissProt, 12 genes were annotated as extracellular regions in GO cellular component (Table 1). Three of the genes responsible for the production of pectinase were observed to be involved in pentose and glucuronate interconversions (ko00040), three genes responsible for cellulase synthesis were also found and which were involved in starch and sucrose metabolism (ko00500), and one gene responsible for in the synthesis of hemicellulose was also identified, which was participated in biosynthesis of secondary metabolites (ko01110). In addition, the results of qRT-PCR validation of these genes were basically consistent with those of RNA-seq (Figure 6).

### 3.7. Analysis of Cell Wall Degrading Enzymes-Related Pathways

**Figure 7:**
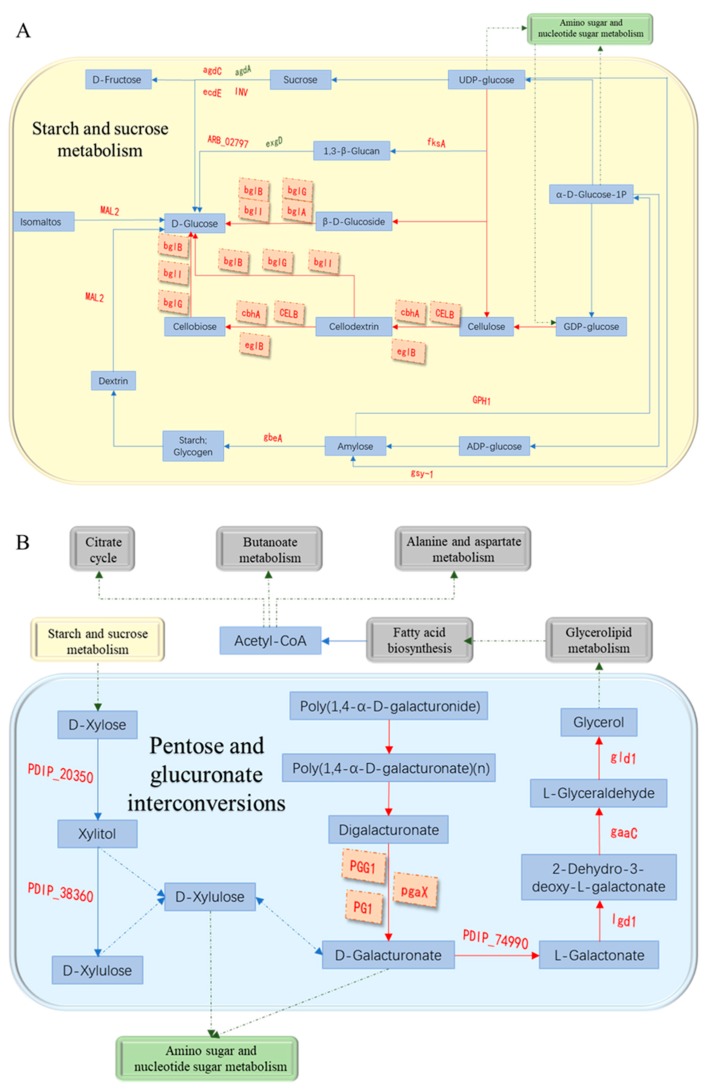
Cell wall degrading enzymes-related pathways. (**A**): Cellulase-related pathways; (**B**): pectinase-related pathways.

After functional annotation of DEGs, we found cellulase-related DEGs—(*PdCELB*, PDIP_47720), (*PdcbhA*, PDIP_71000), (*eglB*(PDIP_65210), (*PdbglB*, PDIP_17570), (*PdbglG*, PDIP_18530) and (*PdbglI*, PDIP_50760) were involved in starch and sucrose metabolism (ko00500) (Figure 7A). They were up-regulated by 40.79, 23.59, 58.49, 6.59, 3.18 and 2.43 times, respectively, and these genes played a key role in the degradation of cellulose and β-D-glucoside into D-glucose. The DEGs associated with pectinase (*PdPG1*, PDIP_64460), (*PdPGG1*, PDIP_19910) and (*PdpgaX*, PDIP_62540) were also found to be involved in pentose and glucuronate interconversions (ko00040) (Figure 7B). They were up-regulated by 237.21, 11.79 times and 22.01 times, respectively, and these genes were indispensable in the process of degradation of poly(1,4-α-D-galacturonide) into D-galacturonate and subsequent conversion to glycerol. Meanwhile, these two pathways were inextricably linked to amino sugar and nucleotide sugar metabolism.

## 4. Discussion

Postharvest green mold decay caused by *P. digitatum* is considered a most alarming disease of citrus [3]. In recent times, several studies have been conducted on conidia growth and the molecular mechanisms of pathogenesis/virulence of the fungus [26]. On the other hand, the specific relationship between the pathogenic fungi and plant/fruit has not been fully elucidated. In this study, early physiological growth of *P. digitatum* in citrus fruits was revealed, combined with the studies that proved the changes/activities of cell wall degrading enzymes in the infection mechanisms of *P. digitatum* in citrus. Moreover, RNA-seq was performed on *P. digitatum* at key times to ascertain the role of cell wall degrading enzymes at the molecular level.

*P. digitatum* lacks the capacity to break through the cuticle on the stratum corneum of citrus surface, so the necessary nutrients for growth must be obtained from the wound. The water-stained soft rot appeared between 36 and 48 h at 25 °C after inoculation of *P. digitatum* spores in the wounds of citrus. The lesion incidence was more than 90% at 48 h and this observation was in agreement with the findings of Vilanova et al., [27]. Results showed that *P. digitatum* spores begun to infect citrus wounds within a short time and begun to colonize. Although *P. digitatum* is the main pathogen of postharvest diseases of citrus, its reproductive cycle is still unclear [26]. Therefore, we tracked the growth/infection of *P. digitatum* spores in vitro and in vivo.

The pathogenic fungus became activated after contact with the host, and the spores gradually expanded after absorbing nutrients [28]. Subsequently, the deposition of polysaccharides such as chitin on the cell wall became polarized, and the extension of fungal cells took place in the restricted area of the cell tip, i.e., germ tube formation [29]. Thereafter, the germ tube gradually grew and eventually formed a branched mycelia. In our experiments, *P. digitatum* spores had been activated and swelled at 6 hpi in vitro, after which it started to form germ tubes. The development of the germ tube was completed between 6 and 9 hpi, and then the germ tube continued to elongate to form a thick branched mycelium before 28 hpi. The phialide structure formed at the end of mycelium 36 hpi, then new conidia were formed on phialide and dropped off at 44 hpi. The results of in vivo growth were consistent with the in vitro studies, however, the interesting part is that the growth of the spores in vivo showed a difference of about 3 h as compared to that in the in vitro growth. It is probably because the spores of *P. digitatum* could directly acquire the carbon source necessary for growth on the PDA, while there is the need to secrete a series of cell wall degrading enzymes from spores to obtain nutrition in citrus fruit wounds [6].

The cell wall of plant is a barrier that must be broken through in the process of infection by pathogenic fungus. Pectin is equally an important component of the layer glue of plant cell wall and plays an important role in the adhesion of cells [12]. In our study, the activity of the two pectinases, PG and PMG, in the wound increased rapidly, and reached its maximum level before and at 48 hpi, respectively (PG reached 3463 U/g, PMG reached 6067 U/g) (Figure 4). This indicates that spores of *P. digitatum* first secreted a series of pectinases to degrade the pectin in the intercellular layer to produce the carbon source necessarily for its growth after invading citrus fruit wounds (Figure 5B). Meanwhile, the lack of pectin causes the softening and decay of citrus wounds which is consistent with the decay incidence of citrus inoculated with *P. digitatum* (Figure 1C). Our research results showed that the activity of cellulase (β-G and Cx) in citrus fruit wounds inoculated with *P. digitatum* did not differ significantly from the control group in the first 48 h compared with pectinases, but their significant differences became prominent after 48 h of inoculation (Figure 4). These results proved that *P. digitatum* further secreted a large amount of cellulase to decompose the cell wall after the softening and relaxation of the tissue (48 h) by pectinase, thereby obtaining a large number of nutrients for its rapid proliferation [30]. This also led to the rapid expansion of the watery soft rot close to the citrus fruit wounds. The appearance of the citrus wound inoculated with *P. digitatum* (Figure 1C) was consistent and corresponds to the soluble sugar content which reached its peak in two days (Figure 5B). Meanwhile, it has been reported that *Botrytis cinerea* secretes oxalic acid to change the host's pH during infection, thus providing a stable environment for CWDEs to function [31]. In this study, the increasing organic acid content after 24 hpi may also be the reason for the rapid increase in CWDE content (Figure 5A). In addition, the decay rate of citrus wounds increased rapidly to 90% within 36–48 hpi (Figure 1A). Various results mentioned above showed that extreme changes were occurred in wounds of *P. digitatum* infected citrus fruits between 36 and 48hpi. Subsequently, on the surface of this softly rotten citrus fruits filled with nutrients, a piece of *P. digitatum* colony that spread out from the wound grew (Figure 1C).

From microscopic observation and physiological study, we determined that the key time point of early infection of *P. digitatum* in citrus is 44 hpi. When RNA-seq was performed on *P. digitatum* before inoculation (from suspension) and after 44 hpi on citrus wounds, the results revealed that expression of genes related to pectinase and cellulase synthesis increased significantly after the spores of *P. digitatum* were inoculated into citrus wounds (Table 1). This is consistent with results of López-Pérez et al. [32]. In addition, the endo-1,4-beta-xylanase-related gene (*Pdxyn2*, PDIP_02920) was up-regulated 21.41 times. It is a cell wall degrading enzyme based on hemicelluloses [33]. It indicated that hemicellulase may also play a role in the *P. digitatum* infection process of citrus. Furthermore, cellulase-related genes were annotated to participate in starch and sucrose metabolism (ko00500) (Figure 7A), which reveals how cellulase participates in the conversion of cellulose into a high-quality carbon source for the growth of *P. digitatum*. It also explained the changes of soluble sugar content in citrus wound during infection at molecular level. On the other hand, pectinase-related genes were annotated to participate in pentose and glucuronate interconversions (ko00040) (Figure 7B). The metabolic pathway indicated that pectinase was involved in the degradation of poly (1,4-α-D-galacturonide) into glycerol (Figure 7B), which can be used as a carbon source for microbial growth [34]. The results also explained the availability of carbon source for *P. digitatum* in the initial infection process in citrus wounds. There might be synthesis of large number of Acetyl-CoA through glycerolipid metabolism and fatty acid biosynthesis. Acetyl-CoA is a necessary participant in the citrate cycle, which is the most critical pathway in almost all organisms [35]. Starch and sucrose metabolism are also related to D-Xylose synthesis which is an important component in pentose and glucuronate interconversions, and indicated that cellulase and pectinase have synergistic effects in the glucose metabolism of *P. digitatum* in infecting citrus. 

In conclusion, the study elucidated the infection mechanism of *P. digitatum* in wound tissues of citrus fruits during the initial infection process by microscopic observation, enzyme activity, physicochemical measurement and comparative transcriptome analysis. In addition, there is still a huge number of data in the RNA-seq that need to be analyzed. There are more DEGs available, which play important role in fungal development and pathogenicity, cause the formation of invasive growth and variation, and produce toxins related to fungal infection of plants which we are interested in. Furthermore, more bioinformatics analysis and functional validation are needed for these genes. Furthermore, considerations on revealing the mechanism of postharvest *P. digitatum* infection in citrus fruits from these analyses will be done, to control the infection. 

## Figures and Tables

**Table 1:** Differentially expressed genes related to cell wall degrading enzymes.

#id	log2 Fold change	Cellular Component (GO^a^)	Swissport annotation (GN^b^)	KEGG^c^ pathway
PDIP_64460	7.89	extracellular region	Polygalacturonase (*PG1*)	Pentose and glucuronate interconversions
PDIP_62540	4.46	extracellular region	exopolygalacturonase X (*pgaX*)	Pentose and glucuronate interconversions
PDIP_19910	3.56	extracellular region	Polygalacturonase (*PGG1*)	Pentose and glucuronate interconversions
PDIP_65210	5.87	extracellular region	endo-beta-1,4-glucanase B (*eglB*)	Starch and sucrose metabolism
PDIP_47720	5.35	extracellular region	Endoglucanase B (*CELB*)	Starch and sucrose metabolism
PDIP_71000	4.56	extracellular region	1,4-beta-D-glucan cellobiohydrolase A (*cbhA*)	Starch and sucrose metabolism
PDIP_17570	2.72	extracellular region	Beta-glucosidase B (*bglB*)	Biosynthesis of secondary metabolites
PDIP_68890	6.00	extracellular region	arabinogalactan endo-beta-1,4-galactanase (*galA*)	
PDIP_55030	4.58	extracellular region	mannan endo-1,4-beta-mannosidase A (*manA*)	
PDIP_02920	4.42	extracellular region	Endo-1,4-beta-xylanase 2 (*xyn2*)	
MSTRG.2424	3.86	extracellular region	Alpha-xylosidase A (*axlA*)	
PDIP_24590	3.73	extracellular region	arabinan endo-1,5-alpha-L-arabinosidase A (*abnA*)	

^a^ Annotation of gene ontology; ^b^ gene name; ^c^ annotation of Kyoto Encyclopedia of Genes and Genomes.
